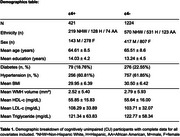# *APOE‐*ɛ4 Moderates the Association between HDL‐c and White Matter Hyperintensity Volume

**DOI:** 10.1002/alz70856_106990

**Published:** 2026-01-08

**Authors:** Zoe E Tsokolas, Amaryllis A Tsiknia, Melissa Petersen, Arthur W. Toga, Hussein N Yassine, Sid E. O'Bryant, Kristine Yaffe, Kevin King, Matthew Borzage, Meredith N. Braskie

**Affiliations:** ^1^ Mark and Mary Stevens Neuroimaging and Informatics Institute, Keck School of Medicine, University of Southern California, Los Angeles, CA, USA; ^2^ Institute for Translational Research, University of North Texas Health Science Center, Fort Worth, TX, USA; ^3^ Center for Personalized Brain Health, Department of Neurology, Keck School of Medicine, University of Southern California, Los Angeles, CA, Los Angeles, CA, USA; ^4^ Weill Institute for Neurosciences, University of California San Francisco, San Francisco, CA, USA; ^5^ Department of Neuroradiology, Barrow Neurological Institute, Phoenix, AZ, USA; ^6^ Fetal and Neonatal Institute, Division of Neonatology, Children's Hospital Los Angeles, Department of Pediatrics, Keck School of Medicine, University of Southern California, Los Angeles, CA, USA; ^7^ Institute for the Developing Mind, Children's Hospital Los Angeles, Los Angeles, CA, USA; ^8^ Department of Psychiatry, Keck School of Medicine, University of Southern California, Los Angeles, CA, USA

## Abstract

**Background:**

Homozygous *APOE‐*ɛ4 carriers have exhibited heightened white matter hyperintensity (WMH) burden based on severity ratings (Rojas et al., 2018). A higher high‐density lipoprotein cholesterol (HDL‐c) to low‐density lipoprotein cholesterol (LDL‐c) ratio is associated with less severe WMHs (Wei et al., 2023). Additionally, higher concentrations of APOE protein are linked to higher cholesterol efflux in Alzheimer's Disease cohorts (Yassine et al., 2016). Despite the understanding of APOE as a lipid carrier (F. Yin, 2021), its mechanistic role in modulating dementia risk is still evolving. Here, we examine whether *APOE‐*ɛ4 positivity modifies the relationship between blood cholesterol levels and WMH volume in a multi‐ethnoracial cohort.

**Method:**

We examined 1645 cognitively unimpaired (CU) individuals from the Health and Aging Brain Study‐Health Disparities cohort (65.96% female, 25.6% *APOE‐*ɛ4+, aged 50‐90) (Table 1). Participants underwent a MR scanning (Siemens 3T Skyra or Vida), which included a T2 FLAIR image. We calculated WMH volume using SPM's lesion growth algorithm (LGA) and ran robust linear regressions to test for an interaction between log‐transformed blood cholesterol levels and *APOE‐*ɛ4 status on log‐transformed WMH volume. Associations between cholesterol markers and WMH volume also were separately examined in *APOE‐*ɛ4 carriers and non‐carriers. Covariates included age, sex, years of education, intracranial volume, MRI scanner, body mass index, diabetes, and hypertension. All continuous variables were standardized. We corrected for three comparisons (HDL‐c, LDL‐c, triglycerides) using the false discovery rate method.

**Result:**

There was a significant HDL‐c×*APOE‐*ɛ4 interaction on WMH volume (β= ‐0.10, *p*‐corrected= 0.03) in the fully‐corrected model driven mainly by a non‐significant association between higher HDL‐c and lower WMH volume in *APOE‐*ɛ4 carriers (β= ‐0.10, *p*‐corrected= 0.17) only. Interactions between *APOE*4 carrier status and LDL‐c and triglycerides were not significant.

**Conclusion:**

*APOE‐*ɛ4 moderated the relationship between HDL‐c and WMH volume such that greater HDL‐c levels were associated with lower WMH burden in ɛ4 carriers only. *APOE‐*ɛ4 did not interact with LDL‐c or triglycerides on WMH volume. This investigation provides support for investigating HDL‐c further in the context of brain health in ɛ4 carriers.